# Polygenic prediction of major depressive disorder and related traits in African ancestries UK Biobank participants

**DOI:** 10.1038/s41380-024-02662-x

**Published:** 2024-07-16

**Authors:** S. C. Kanjira, M. J. Adams, Y. Jiang, C. Tian, 23andMe Research Team, Y. Jiang, C. Tian, C. M. Lewis, K. Kuchenbaecker, A. M. McIntosh

**Affiliations:** 1 Centre for Clinical Brain Sciences, University of Edinburgh, Edinburgh, UK; 2 Malawi Epidemiology and Intervention Research Unit, Lilongwe, Malawi; 3 23andMe Inc, Sunnyvale, CA USA; 4 Social, Genetic and Developmental Psychiatry Centre, Institute of Psychiatry, Psychology & Neuroscience, King’s College London, London, UK; 5 UCL Genetics Institute, University College London, London, UK; 6 Centre for Genomic and Experimental Medicine, University of Edinburgh, Edinburgh, UK

**Keywords:** Depression, Genetics

## Abstract

Genome-Wide Association Studies (GWAS) over-represent European ancestries, neglecting all other ancestry groups and low-income nations. Consequently, polygenic risk scores (PRS) more accurately predict complex traits in Europeans than African Ancestries groups. Very few studies have looked at the transferability of European-derived PRS for behavioural and mental health phenotypes to Africans. We assessed the comparative accuracy of depression PRS trained on European and African Ancestries GWAS studies to predict major depressive disorder (MDD) and related traits in African ancestry participants from the UK Biobank. UK Biobank participants were selected based on Principal component analysis clustering with an African genetic similarity reference population, MDD was assessed with the Composite International Diagnostic Interview (CIDI). PRS were computed using PRSice2 software using either European or African Ancestries GWAS summary statistics. PRS trained on European ancestry samples (246,363 cases) predicted case control status in Africans of the UK Biobank with similar accuracies (R2 = 2%, β = 0.32, empirical p-value = 0.002) to PRS trained on far much smaller samples of African Ancestries participants from 23andMe, Inc. (5045 cases, R² = 1.8%, β = 0.28, empirical p-value = 0.008). This suggests that prediction of MDD status from Africans to Africans had greater efficiency relative to discovery sample size than prediction of MDD from Europeans to Africans. Prediction of MDD status in African UK Biobank participants using GWAS findings of likely causal risk factors from European ancestries was non-significant. GWAS of MDD in European ancestries are inefficient for improving polygenic prediction in African samples; urgent MDD studies in Africa are needed.

## Introduction

Depressive disorders are ranked as the third leading cause of disability, as measured by years lived with disability, with Major Depressive Disorder (MDD) being the most significant contributor to this burden. The World Health Organization estimates that more than 322 million individuals globally suffer from MDD, with at least 9% of these cases occurring in Africa [1, 2]. While lower rates of MDD have been reported in Africa compared to Europe and North America, recent studies suggest that MDD is under-reported in Africa and that most affected individuals go undiagnosed [3, 4].

MDD has a heritability of 30–40% [5] and better characterisation of its genetic architecture may provide both an improved mechanistic understanding and more accurate genetic prediction. So far, genome wide association studies (GWAS) have successfully identified over 243 variants to be associated with depression, focussing on participants of European ancestry [6]. Sirugo et al. showed that GWAS studies overrepresent European compared to other ancestry groups, with an approximately fivefold over-representation compared to their global population [7–9]. The overrepresentation of Europeans in genetics research means that the potential benefits of these studies will disproportionately apply to people of European ancestry and deprive other ancestries and low-income countries of new treatments and diagnostics [10].

Due to overrepresentation of Europeans in GWAS, polygenic risk scores (PRS) developed from these studies more accurately predict many complex traits in European than in African Ancestries samples [11, 12]. The difference in prediction may be due to differences in the phenotypes themselves, their genetic architectures or because of gene-by-environment interactions [13–15]. Very few studies have looked at the transferability of European-derived PRS for behavioural and mental health phenotypes to non-Europeans generally and Africans specifically. Consequently, the predictive accuracy of European derived depression PRS to African samples remains uncertain. We looked at the transferability of MDD-PRS trained on European GWAS studies to African Ancestries participants from the UK Biobank within and across traits. Furthermore, we sought to compare the transferability of MDD-PRS trained in participants of African ancestries from 23andMe Inc., (mainly from North America), with the transferability of MDD-PRS trained on Europeans to the African-ancestry participants from the UK Biobank.

## Methodology

### Samples

The study focused on African participants in the UK Biobank who have a shared genetic similarity with 1000 Genomes Project’s African reference samples. The UK Biobank is a prospective cohort study of individuals of diverse ethnic backgrounds from across the United Kingdom [16]. We used Principal component analysis (PCA) to identify these participants of African ancestral background within the UK Biobank. Participants were initially selected based on self-report, individuals who self-reported as being Black or Black British (Caribbean, African, Any other Black background), White and Black Caribbean or Black African, and participants whose self-identity was not specifically categorised (responses “Other ethnic group”, “Any other mixed background”, “Do not know”, or “Prefer not to answer’) were selected. Using the genotypes provided by UK Biobank, we derived ancestry informative genetic principal components using the weights from the 1000 Genomes reference dataset to cluster the participants into their genetic similarity groupings. UK Biobank participants who clustered closely with the 1000 Genomes African (AFR) reference group were then selected for further analysis.

An online Mental Health Questionnaire that included a depression assessment was sent to UK Biobank participants by email and entitled ‘The thoughts and feelings questionnaire’ [17]. The questionnaire was offered to the 317,785 participants, out of the total 502,616 UK Biobank participants, who had agreed to email contact, and 157,396 completed the online questionnaires by June 2018, of these 1090 participants were of African ancestry. A depression phenotype was generated based on the CIDI-SF (Composite International Diagnostic Interview Short Form) [18]. Cases were defined as those participants who had at least one core symptom of depression (persistent sadness or loss of interest) for most of the day or all of the day. Symptoms had to be present for a period of over two weeks plus another four non-core depressive symptoms that represent a change from usual occurring over the same timescale, with some or a lot of impairment. Cases that self-reported another mood disorder were excluded. Controls were defined as participants who did not meet symptom criteria for MDD [17, 19].

#### Polygenic risk scores

Polygenic risk scores were computed using PRSice-2 software [20]. PRSice2 software uses the clumping and thresholding method (C + T) to retain only SNPs that are weakly correlated with one another [20]. After clumping, SNPs with a p value larger than a specified level of significance were removed, PRS were then calculated by the sum of SNP allele effect sizes multiplied by the number of risk alleles. Both the base and target data sets were quality controlled (QC) by removing ambiguous and duplicate SNPs, SNPs with a minor allele frequency (MAF) of less than 1% and a genotype missingness greater than 2% were also removed. We report standardised effect sizes. Additionally, we provide empirical P values after specifying 10,000 permutations in PRSice-2.

#### Summary statistics

To compute polygenic risk scores in African-clustered participants of the UK Biobank, we used GWAS summary statistics of depression from global European studies (246,363 cases and 561,190 controls), a predominantly African American study from 23andMe (5045 cases, 102 098 controls), a secondary dataset comprising summary statistics from a meta-analysis of 12 African cohorts (36,313 cases, 160,775 control) and data from several traits that have been known to be associated with depression from European-clustered studies, in addition to height, which we used as a negative control. All summary statistics used in this study have been shown in Table 1, for each set of summary statistics, SNP based heritability was calculated using linkage disequilibrium score regression as implemented in the LDSC software package [21, 22]. To calculate heritability with LDSC, we employed LD Scores derived from UK Biobank data. Specifically, we used European LD Scores for European datasets and African LD Scores for African datasets, both sourced from the UK Biobank.

**Table 1:** Summary Statistics Used in PRS Analysis.

Phenotype	Ancestry group	N Cases	N controls	h^2^_SNP_ (%)	Reference
Major depression	European	246,363	561,190	6.68	Howard et al. [28]
Major depression	Predominantly African American	5045	102,098	−0.72	23andMe, unpublished
Major depression	Meta-analysed African data	36,313	160,775	2.77	Meng et al. 2022 (unpublished)
BMI	European	339,224	–	12.21	Locke et al. [29]
Height	European	4,080,687	–	38.47	Yengo et al. [30]
Schizophrenia	European	67,390	94,015	16.22	Trubetskoy et al. [31]
Educational attainment	European	765,283	–	12.11	Okbay et al. [32]
Bipolar disorder	European	41,917	371,549	9.86	Mullins et al. [33]
Neuroticism	European	390,278	—	2.71	Nagel et al. [34]

Summary statistics used in PRS analysis, detailing source, ancestry group, number of cases and controls, and SNP-based Heritability for each study.

## Results

### African-ancestries clustered participants in the UK Biobank

**Fig. 1:**
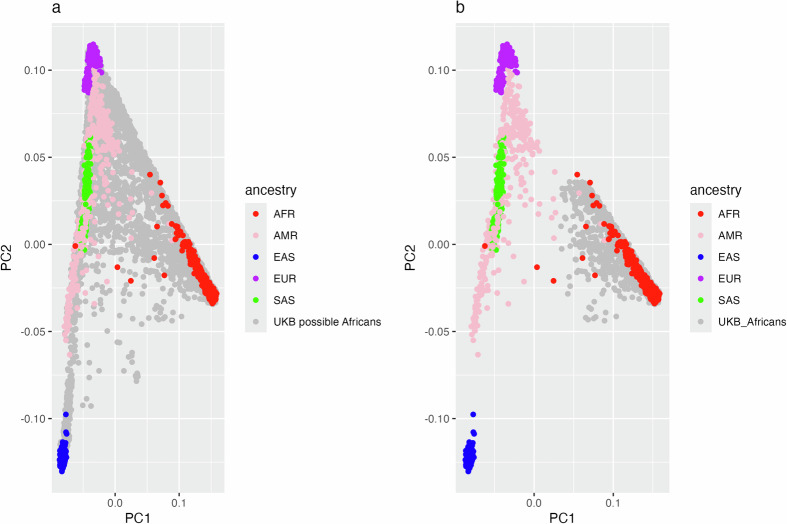
PCA Plots of UK Biobank participants and 1000 Genomes reference dataset: self-reported and genetically inferred African ancestry. **a** A PCA plot showing the clustering of individuals of possible African ancestry (people who self-reported as being Black or Black British, other ethnic grouping, do not know, mixed, and prefer not to answer) in the UK Biobank with the reference 1000 Genomes dataset. Different colours represent the different ancestry groups in the reference 1000 Genomes dataset (AFR Africans, AMR Americans, EAS Asians, EUR Europeans, and SAS South Asians), while the grey colour indicates individuals of possible African ancestry based on self-report from the UK Biobank. **b** A PCA plot showing UK Biobank participants(grey) selected to be of African ancestry using PCA, alongside the reference 1000 Genomes participants.

We utilised principal component analysis (PCA) to project the genetic data of UK Biobank participants onto the PCA space defined by the reference 1000 Genomes dataset. This approach enabled the identification of individuals from the UK Biobank whose genetic profiles closely resemble those of the African ancestry samples within the 1000 Genomes dataset Fig. 1.

UK Biobank participants of possible African ancestry that clustered within 6 standard deviations of the 1000 Genomes AFR cluster were selected to represent Africans in the UK Biobank. In total, 8543 participants in the UK Biobank clustered with the 1000 Genomes AFR reference group. Of these, 1090 participants completed the Mental Health Questionnaire, with 190 participants meeting the CIDI criteria for MDD cases and 671 participants meeting the criteria for controls.

### Polygenic risk scores

#### Within ancestry within trait polygenic prediction of MDD from African datasets to African participants in the UK Biobank

**Fig. 2:**
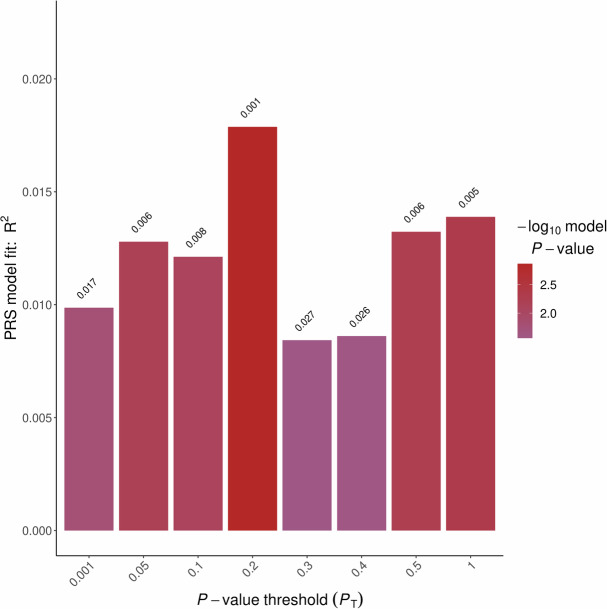
Prediction of MDD in African participants of the UK Biobank using predominantly African American data (5045 cases and 102 098 controls) from 23andMe. The y-axis displays the R2 values corresponding to the PRS predictions, while the x-axis illustrates the spectrum of p-value thresholds utilised in the PRS analysis. The p-values for the predictions are depicted atop the bars.

Depression GWAS results of African participants from 23andMe (5045 cases and 102 098 controls) were used to predict MDD status in African participants of the UK Biobank (see Fig. 2). The summary statistics from 23andMe African ancestry significantly predicted MDD status in UK Biobank African participants across all P-value thresholds, with the most predictive P-value threshold being 0.2, explaining 1.8% of variation in MDD liability. The prediction was associated with a beta coefficient of 0.28(SE = 0.08, empirical P-value = 0.008). This PRS prediction of MDD from African sample to African sample is comparable in accuracy with prediction of PRS trained on European ancestry samples of over 800 K individuals (246,363 cases and 561,190 controls).

**Fig. 3:**
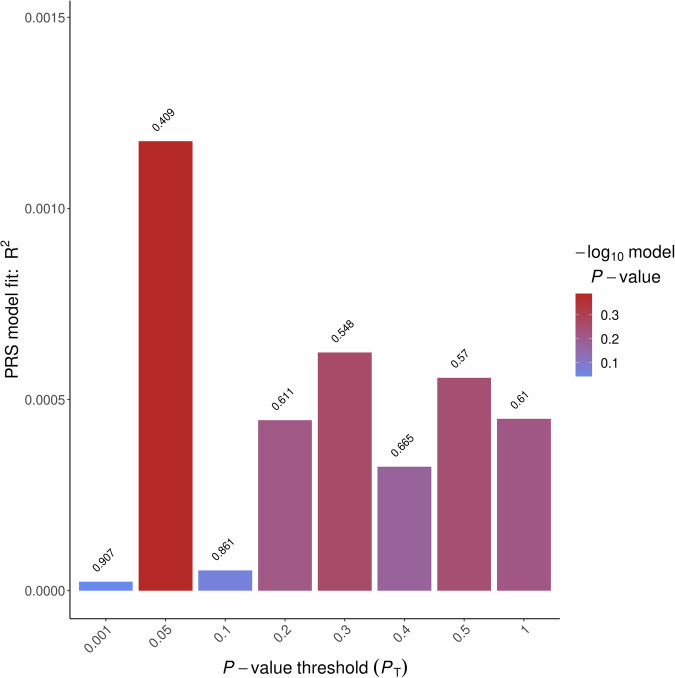
Prediction of MDD status in African participants of UK Biobank using meta-analysed African MDD summary statistics from multiple African American (99%) and one Africa-based study.

We also used a secondary set of summary statistics from a meta-analysed data of 12 African cohorts with 36,313 depression cases and 160 775 controls to predict MDD status in African participants of the UK Biobank. 99.6% of the cases in this meta-analysed dataset are multiple African American studies and 0.4% participants are from South Africa. In contrast, PRS trained on the meta-analysed African American dataset *did not* significantly predict MDD status in Africans of the UK Biobank, as shown in Fig. 3.

#### Cross ancestry within trait prediction of MDD and other traits

**Fig. 4:**
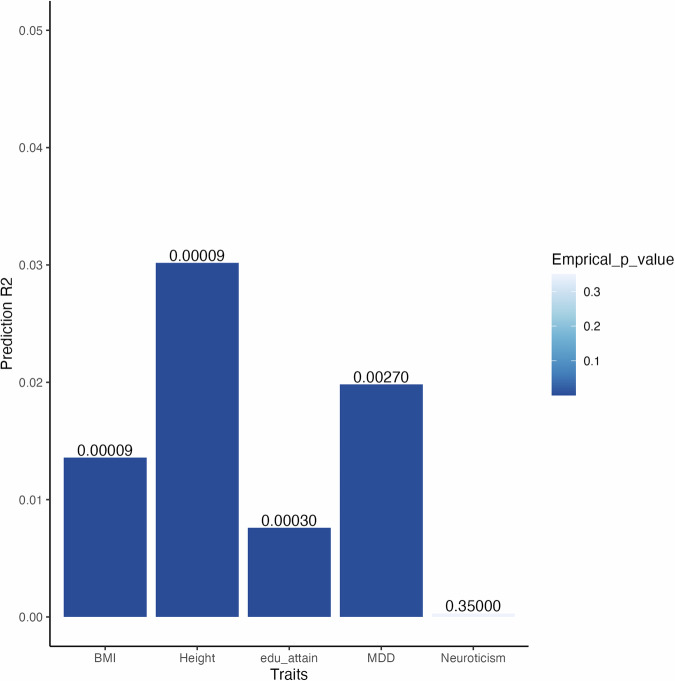
Bar plots showing cross-ancestry within trait polygenic prediction of BMI, height, education attainment (edu_attain), MDD and Neuroticism. The y-axis is showing the R^2^ values for the PRS predictions while the x-axis is showing the most predictive bar for each trait with empirical P values for the prediction shown on top of the bars.

European-based GWAS results for depression, BMI, Neuroticism, education attainment and height were used to predict within the same trait in UK Biobank African Ancestries participants. PRS trained on European GWAS results significantly predicted MDD, BMI, education attainment, and height within trait in African participants of the UK Biobank as illustrated in Fig. 4.

Specifically, the European based depression PRS explained a 2% variation in MDD risk among individuals of African Ancestries in the UK Biobank, with a beta coefficient of 0.32(SE = 0.09, empirical P-value = 0.002). The PRS associated with education attainment explained 0.7% of the variation in education attainment within the same cohort, this prediction had a beta coefficient equal 0.079 and a P-value = 0.0003 (Fig. 4). However, it is noteworthy that European-based Neuroticism PRS did not significantly predict Neuroticism in African Ancestries participants. Height PRS, a highly heritable trait used for comparison purposes, explained 3% of the variation in height among UK Biobank Africans.

#### Cross ancestry cross trait polygenic prediction of MDD

**Fig. 5:**
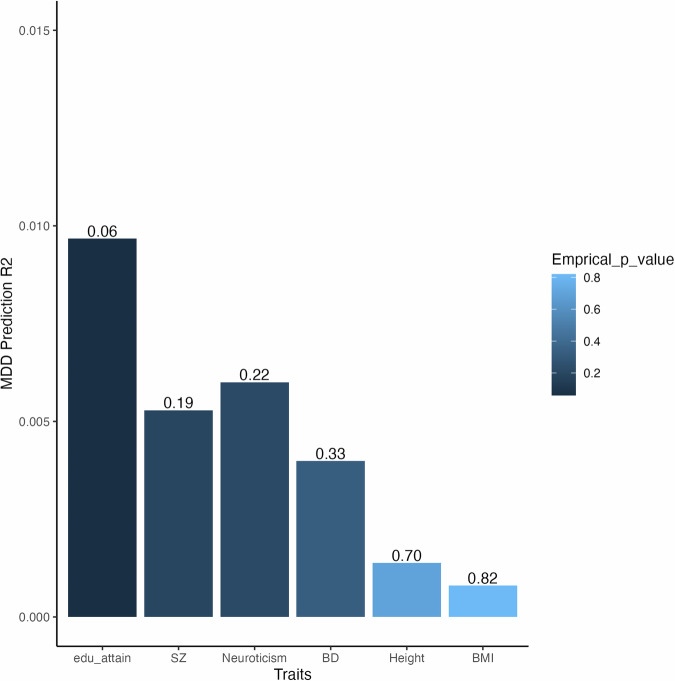
Bar plot of cross ancestry cross trait polygenic prediction of MDD using Education attainment (edu_attain), Schizophrenia (SZ), bipolar disorder (BD), Neuroticism, Height, and BMI. The y-axis displays the R^2^ values for the PRS predictions, while the x-axis represents the most predictive bar associated with each trait, and the empirical P values for the predictions are presented atop these bars.

Cross ancestry cross trait polygenic prediction of MDD in Africans of the UK Biobank using PRS estimated from European based GWAS summary statistics for traits known to be associated with MDD (namely: Bipolar Disorder, BMI, Schizophrenia, Neuroticism, and education attainment) did not show any significant association (see Fig. 5). Height summary statistics were used for comparison purposes.

## Discussion

Polygenic risk scores trained on European GWAS studies of 246,363 depression cases predicted MDD case-control status in African Ancestries participants from UK Biobank at lower prediction accuracies (R^2^ = 2%) than for European participants (R^2^ = 3.2%). Polygenic risk scores trained on a much smaller single GWAS of 5045 depression cases from 23andMe Africans predicted depressed case-control status in African Ancestries participants from UK Biobank at a similar accuracy (R^2^ = 1.8%), suggesting a much greater prediction accuracy relative to sample size from African than from European GWAS training datasets.

While we acknowledge that the 23andMe summary statistics used in our study indicated a negative heritability, the estimation of heritability in small sample sizes can be methodologically challenging. Large African population datasets and LD reference panels are scarce, and these findings further underpin the need for expanding research in underrepresented populations. Nevertheless, the observation that we were able to predict from the 23andMe African ancestry samples into an independent cohort suggests that MDD-relevant information is contained within these genetic associations.

Prediction of MDD status in African UK Biobank participants using European ancestry GWAS studies of other traits (known to predict MDD status in European populations) was non-significant. GWAS studies of depression from European samples are an inefficient means of improving polygenic prediction accuracy in African samples and genetic scores derived from European ancestry studies of known risk factors for depression may also be less useful for mechanistic studies.

In contrast to the findings made using 23andMe summary statistics, polygenic scores derived from a GWAS meta-analysis of several African ancestry studies within and outside of Africa, showed limited predictive ability for Major Depressive Disorder (MDD) in African Ancestries UK Biobank participants. The dataset combined 36,313 MDD cases and 160 775 controls, predominantly comprising African Americans, with a small representation (139 cases and 346 controls) from continental African populations (Drakenstein Child Health Study). Despite expectations of superior performance compared to the 23andMe dataset, various factors may have contributed to this underperformance. Firstly, while 23andMe used a single definition of MDD and a single genotyping quality-control pipeline, MDD phenotype definitions and methods varied across the included cohorts in the GWAS meta-analysis. Some studies employed stringent criteria while others used broader definitions. The inclusion of individuals with varying definitions of African Ancestries may also have increased genetic heterogeneity. The meta-analysed data primarily featured African Americans, who exhibit varying degrees of genetic admixture with other ancestral backgrounds, possibly influencing the accuracy of PRS predictions in UK Biobank. A recent study by Ding et al. in 2023 revealed that for highly polygenic traits, PRS predictive accuracy tends to diminish with increasing genetic distance between populations [23].

Several European ancestry studies have shown that various traits have a shared genetic liability with MDD, some of which may be causally associated but little is known about the shared genetic liability of MDD with other traits across ancestries [24]. We looked at cross ancestry cross trait prediction of MDD using height, BMI, bipolar disorder, schizophrenia, neuroticism, and education attainment in people of African Ancestries using European based GWAS results. Height was used as a highly heritable control trait with no known causal relationship with MDD. While our study did not yield successful predictions of MDD status in Africans of the UK Biobank using European GWAS results of various traits, it is worth noting that previous investigations conducted within European populations have demonstrated a shared genetic liability between MDD and traits such as Bipolar Disorder, BMI, and neuroticism [25–27]. To advance our understanding of shared genetic liability in African populations, future research endeavours could explore this aspect by training PRS using GWAS data derived specifically from African cohorts. This approach has the potential to uncover novel insights into the shared genetic components between MDD and other traits within the context of African ancestral backgrounds.

The predictive accuracy of MDD-PRS trained on predominantly African American data from 23andMe was broadly comparable to that of PRS trained on European data among individuals of African Ancestries, despite the African American PRS being based on a considerably smaller sample size compared to the European PRS. This observation aligns with findings from other studies focused on various traits, suggesting that PRS derived from African Ancestries data tends to exhibit superior performance when applied to African populations than PRS derived from European data.

## Supplementary information

23andMe Research Team

## Data Availability

Access to UK Biobank data can be obtained by following the procedure described at http://www.ukbiobank.ac.uk/using-the-resource/. European MDD summary statistics from Howard et al. [28] are available from 10.7488/ds/2458. BMI and height summary statistics are available from the GIANT consortium. Schizophrenia, Bipolar disorder, depression and Neuroticism summary statistics are publicly available from the Psychiatric Genomics Consortium (PGC) https://pgc.unc.edu/for-researchers/download-results/. The full GWAS summary statistics for the 23andMe data set are available to qualified researchers through 23andMe under an agreement. For more information and to apply for access, visit the 23andMe research website: https://research.23andme.com/collaborate/.

## Code availability

We used publicly available software PRSice-2 and its code is publicly available at https://github.com/choishingwan/PRSice. The tutorial for using PRSice2 is available at https://choishingwan.github.io/PRS-Tutorial/prsice/.

## References

[1] James SL, Abate D, Abate KH, Abay SM, Abbafati C, Abbasi N, et al. Global, regional, and national incidence, prevalence, and years lived with disability for 354 diseases and injuries for 195 countries and territories, 1990–2017: a systematic analysis for the Global Burden of Disease Study 2017. Lancet. 2018;392:1789–858. 30496104 10.1016/S0140-6736(18)32279-7 PMC6227754

[2] World Health Organization. Depression and other common mental disorders: global health estimates. Geneva: World Health Organization; 2017. Report No. WHO/MSD/MER/2017.2. https://apps.who.int/iris/handle/10665/254610

[3] Fekadu A, Demissie M, Berhane R, Medhin G, Bitew T, Hailemariam M, et al. Under detection of depression in primary care settings in low and middle-income countries: a systematic review and meta-analysis. Syst Rev. 2022;11:21. 35123556 10.1186/s13643-022-01893-9 PMC8818168

[4] Rathod S, Roberts T, Medhin G. Detection and treatment initiation for depression and alcohol use disorders: facility-based cross-sectional studies in five low-income and middle-income country districts. BMJ Open. 2018;8:e023421. 30309992 10.1136/bmjopen-2018-023421 PMC6252626

[5] Sullivan PF, Neale MC, Kendler KS. Genetic epidemiology of major depression: review and meta-analysis. Am J Psychiatry. 2000;157:1552–62. 11007705 10.1176/appi.ajp.157.10.1552

[6] Als TD, Kurki MI, Grove J, Voloudakis G, Therrien K, Tasanko E, et al. Depression pathophysiology, risk prediction of recurrence and comorbid psychiatric disorders using genome-wide analyses. Nat Med. 2023;29:1832–44. 37464041 10.1038/s41591-023-02352-1 PMC10839245

[7] Duncan L, Shen H, Gelaye B, Meijsen J, Ressler K, Feldman M, et al. Analysis of polygenic risk score usage and performance in diverse human populations. Nat Commun. 2019;10:3328. 31346163 10.1038/s41467-019-11112-0 PMC6658471

[8] Martin AR, Kanai M, Kamatani Y, Okada Y, Neale BM, Daly MJ. Clinical use of current polygenic risk scores may exacerbate health disparities. Nat Genet. 2019;51:584–91. 30926966 10.1038/s41588-019-0379-x PMC6563838

[9] Sirugo G, Williams SM, Tishkoff SA. The missing diversity in human genetic studies. Cell. 2019;177:26–31. 30901543 10.1016/j.cell.2019.02.048 PMC7380073

[10] Bentley AR, Callier S, Rotimi CN. Diversity and inclusion in genomic research: why the uneven progress? J Community Genet. 2017;8:255–66. 28770442 10.1007/s12687-017-0316-6 PMC5614884

[11] Fritsche LG, Ma Y, Zhang D, Salvatore M, Lee S, Zhou X, et al. On cross-ancestry cancer polygenic risk scores. PLOS Genet. 2021;17:e1009670. 34529658 10.1371/journal.pgen.1009670 PMC8445431

[12] Mars N, Kerminen S, Feng YCA, Kanai M, Läll K, Thomas LF, et al. Genome-wide risk prediction of common diseases across ancestries in one million people. Cell Genomics. 2022;2:100118. 35591975 10.1016/j.xgen.2022.100118 PMC9010308

[13] Petrovski S, Goldstein DB. Unequal representation of genetic variation across ancestry groups creates healthcare inequality in the application of precision medicine. Genome Biol. 2016;17:157. 27418169 10.1186/s13059-016-1016-y PMC4944427

[14] Timpson N, Greenwood C, Soranzo N. Genetic architecture: the shape of the genetic contribution to human traits and disease. Nat Rev Genet. 2017;18:110–24. 10.1038/nrg.2017.101 29225335

[15] Wojcik GL, Graff M, Nishimura KK, Tao R, Haessler J, Gignoux CR, et al. Genetic analyses of diverse populations improves discovery for complex traits. Nature. 2019;570:514–8. 31217584 10.1038/s41586-019-1310-4 PMC6785182

[16] Bycroft C, Freeman C, Petkova D, Band G, Elliott LT, Sharp K, et al. The UK Biobank resource with deep phenotyping and genomic data. Nature. 2018;562:203–9. 30305743 10.1038/s41586-018-0579-z PMC6786975

[17] Davis KAS, Coleman JRI, Adams M, Allen N, Breen G, Cullen B, et al. Mental health in UK Biobank – development, implementation and results from an online questionnaire completed by 157 366 participants: a reanalysis. BJPsych Open. 2020;6:e18. 32026800 10.1192/bjo.2019.100 PMC7176892

[18] Kessler RC, Andrews G, Mroczek D, Ustun B, Wittchen HU. The World Health Organization Composite International Diagnostic Interview short-form (CIDI-SF). Int J Methods Psychiatr Res. 1998;7:171–85.

[19] Howard DM, Folkersen L, Coleman JRI, Adams MJ, Glanville K, Werge T, et al. Genetic stratification of depression in UK Biobank. Transl Psychiatry. 2020;10:163. 32448866 10.1038/s41398-020-0848-0 PMC7246256

[20] Choi SW, O’Reilly PF. PRSice-2: Polygenic Risk Score software for biobank-scale data. GigaScience. 2019;8:giz082. 31307061 10.1093/gigascience/giz082 PMC6629542

[21] Bulik-Sullivan, Loh BK, Finucane PR, Ripke HK, Yang S, Schizophrenia Working Group of the Psychiatric Genomics Consortium J, et al. LD Score regression distinguishes confounding from polygenicity in genome-wide association studies. Nat Genet. 2015;47:291–5. 25642630 10.1038/ng.3211 PMC4495769

[22] Finucane HK, Bulik-Sullivan B, Gusev A, Trynka G, Reshef Y, Loh PR, et al. Partitioning heritability by functional annotation using genome-wide association summary statistics. Nat Genet. 2015;47:1228–35. 26414678 10.1038/ng.3404 PMC4626285

[23] Ding Y, Hou K, Xu Z, Pimplaskar A, Petter E, Boulier K, et al. Polygenic scoring accuracy varies across the genetic ancestry continuum. Nature. 2023;618:774–81. 37198491 10.1038/s41586-023-06079-4 PMC10284707

[24] Edwards AC, Docherty AR, Moscati A, Bigdeli TB, Peterson RE, Webb BT, et al. Polygenic risk for severe psychopathology among Europeans is associated with major depressive disorder in Han Chinese women. Psychol Med. 2018;48:777–89. 28969721 10.1017/S0033291717002148 PMC5843532

[25] Adams MJ, Howard DM, Luciano M, Clarke TK, Davies G, Hill WD, et al. Genetic stratification of depression by neuroticism: revisiting a diagnostic tradition. Psychol Med. 2020;50:2526–35. 31576797 10.1017/S0033291719002629 PMC7737042

[26] Bahrami S, Steen NE, Shadrin A, O’Connell K, Frei O, Bettella F, et al. Shared genetic loci between body mass index and major psychiatric disorders. JAMA Psychiatry. 2020;77:1–11. 10.1001/jamapsychiatry.2019.4188 PMC6990967 31913414

[27] Power RA, Tansey KE, Buttenschøn HN, Cohen-Woods S, Bigdeli T, Hall LS, et al. Genome-wide association for major depression through age at onset stratification: Major Depressive Disorder Working Group of the Psychiatric Genomics Consortium. Biol Psychiatry. 2017;81:325–35. 27519822 10.1016/j.biopsych.2016.05.010 PMC5262436

[28] Howard DM, Adams MJ, Clarke TK, Hafferty JD, Gibson J, Shirali M, et al. Genome-wide meta-analysis of depression identifies 102 independent variants and highlights the importance of the prefrontal brain regions. Nat Neurosci. 2019;22:343–52. 30718901 10.1038/s41593-018-0326-7 PMC6522363

[29] Locke AE, Kahali B, Berndt SI, Justice AE, Pers TH, Day FR, et al. Genetic studies of body mass index yield new insights for obesity biology. Nature. 2015;518:197–206. 25673413 10.1038/nature14177 PMC4382211

[30] Yengo L, Vedantam S, Marouli E, Sidorenko J, Bartell E, Sakaue S, et al. A saturated map of common genetic variants associated with human height. Nature. 2022;610:704–12. 36224396 10.1038/s41586-022-05275-y PMC9605867

[31] Trubetskoy V, Panagiotaropoulou G, Awasthi S, Braun A, Kraft J, Skarabis N, et al. Mapping genomic loci implicates genes and synaptic biology in schizophrenia. Nature. 2022;604:502–8. 35396580 10.1038/s41586-022-04434-5 PMC9392466

[32] Okbay A, Wu Y, Wang N, Jayashankar H, Bennett M, Nehzati SM, et al. Polygenic prediction of educational attainment within and between families from genome-wide association analyses in 3 million individuals. Nat Genet. 2022;54:437–49. 35361970 10.1038/s41588-022-01016-z PMC9005349

[33] Mullins N, Forstner AJ, O’Connell KS, Coombes B, Coleman JRI, Qiao Z, et al. Genome-wide association study of over 40,000 bipolar disorder cases provides new insights into the underlying biology. Nat Genet. 2021;53:817–29. 34002096 10.1038/s41588-021-00857-4 PMC8192451

[34] Nagel M, Jansen PR, Stringer S, Watanabe K, de Leeuw CA, Bryois J, et al. Meta-analysis of genome-wide association studies for neuroticism in 449,484 individuals identifies novel genetic loci and pathways. Nat Genet. 2018;50:920–7. 29942085 10.1038/s41588-018-0151-7

